# Geant4 Simulation of Photon- and Neutron-Shielding Capabilities of Biopolymer Blends of Poly(lactic acid) and Poly(hydroxybutyrate)

**DOI:** 10.3390/polym15214257

**Published:** 2023-10-29

**Authors:** Hanan Akhdar, Maryam Alshehri

**Affiliations:** Department of Physics, Faculty of Science, Imam Mohammad Ibn Saud Islamic University (IMSIU), P.O. Box 90950, Riyadh 11623, Saudi Arabia; mfalshehri@imamu.edu.sa

**Keywords:** biopolymer blends, PLA, PHB, photon, neutron, shielding, Geant4

## Abstract

Simulation is used by scientists to imitate a real-life experimental setup in order to save time, costs and effort. Geant4, a toolkit based on the Monte Carlo method, has been widely used in investigating the radiation-shielding properties of different materials. In many recent studies, researchers have focused on polymers and their shielding capabilities. Poly(lactic acid) (PLA) is a widely used biopolymer in many applications due to its excellent mechanical properties. However, it has limitations related to its degree of crystallinity and molecular characteristics, which could be improved through blending with other biodegradable polymers such as poly(hydroxybutyrate) (PHB). Previous published studies have shown that the mechanical properties of such blends can be improved further. In this work, the effect of blending PHB with PLA on the photon- and neutron-shielding capabilities will be investigated using Geant4 over a wide energy range, as well as the effect of doping those blends with metal oxides. The results show that the shielding properties of the polymers are affected by blending with other polymers and by doping the polymer blends with different metal oxides, and they confirm that Geant4 is a very reliable tool that can simulate any material’s shielding properties against photons and neutrons.

## 1. Introduction

Biopolymers have been receiving more attention recently due to the environmental concerns in many applications. One of the most widely studied biopolymers is polylactic acid (PLA) because it is known for its availability, low cost, high transparency, thermal plasticity and good mechanical properties [1,2,3,4]. It is also biodegradable and biocompatible and can be produced from renewable resources, with properties that are very close to those of some synthetic fossil-fuel-based polymers. PLA plays an important role in many applications, ranging from the industrial to biomedical fields [5,6,7].

Despite the values of tensile strength and elastic modulus and the properties that make PLA very similar to high-performance polymers, it has some drawbacks, such as its brittleness and poor barrier properties, which restrict its range of application [8,9,10,11,12]. 

One of the strategies proposed to overcome those drawbacks is the formulation of PLA-based polymer blends. Recent studies have shown that a suitable polymer for PLA-based blend formation is poly (3-hydroxybutyrate) (PHB). PHB is a biodegradable thermoplastic that plays a role in many industrial applications [13,14,15,16,17,18,19]. PLA–PHB blends have been the focus of recent research because the combination of both biopolymers enhances their mechanical and tensile properties, making them more desirable in a wide range of applications [20,21,22,23,24,25,26,27]. Another strategy to enhance polymers’ properties is to dope them with metal oxides. Many research works have studied the effect of different dopants on the optical and electrical properties of polymers. Recent research has avoided using lead oxides and focused instead on nontoxic metal oxides [28,29,30].

Radiation plays an important role in our lives, but it can cause damage either directly or indirectly by penetrating human bodies [31,32,33,34]. This is why researchers are constantly studying the radiation-shielding capabilities of materials used in industry and medicine, and polymers are receiving more attention in the radiation-shielding field because of their mechanical properties and their abilities to shield against radiation [3,4,5,6,7,8,9,10,11,12,13,14,15,16,17,18,19,20,21,22,23,24,25,26,27,28,29,30,31,32,33,34,35,36,37,38,39,40].

A recent study showed that doping Poly(lactic acid) (PLA)/Poly(ethylene glycol) (PEG) blended films with different amounts of lead increased their gamma-shielding abilities [41]. 

In this work, PLA–PHB blends were studied in terms of photon- and neutron-shielding capabilities. The shielding properties of pure PLA were compared to that of PLA-based blends with PHB at different percentages (10, 20 and 30%). The photon and neutron attenuation coefficients of the studied blends were theoretically investigated at a large energy range (from 10 to 2000 keV) using the Monte Carlo simulation toolkit Geant4. 

Then, the PLA-based blends with PHB at different percentages (10–30%) were doped with 10% of different metal oxides, namely, zinc oxide (ZnO), iron oxide (Fe_2_O_3_), titanium oxide (Ti_2_O) and magnesium oxide (MgO), and their effects on the photon- and neutron-shielding capabilities were also investigated using Geant4. The novelty of this work comes from studying the shielding abilities of the PLA/PHB blends and from doping the blends with metal oxides, which is presented theoretically using Geant4. 

The Geant4 simulation reduced the time and cost needed to make such an investigation. It also enabled the study of different percentages at different energies and the effect of different dopants on the shielding capabilities of the studied polymer blends. 

## 2. Materials 

**Figure 1:**
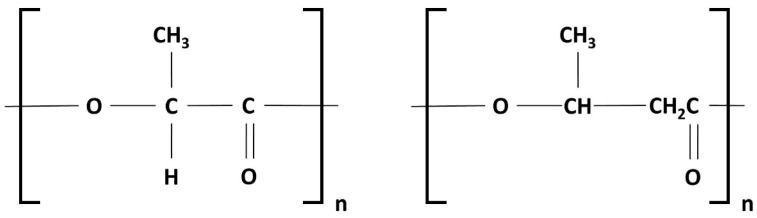
Repeating unit of PLA (**left**) and PHB (**right**) polymers.

PLA and PHB are both biopolymers. The density of PLA is 1.24 g/cm^3^, and the repeating unit is (C_3_H_4_O_2_)_n_, while the density of PHB is 1.19 g/cm^3^ and the repeating unit is (C_4_H_6_O_2_)_n_, as shown in Figure 1. Table 1 summarizes the element weight fractions of both biopolymers [24]. The influence of blending the high-molecular-weight PHB with the low-molecular-weight PLA on the attenuation of the blends against radiation was studied. 

## 3. Theory

### 3.1. Photon Attenuation

The photon mass attenuation coefficient of any material can be calculated using Equation (1) [42,43,44,45]:

(1) $$I=I_{0}e^{-\mu x}$$

When photons enter a material of thickness *x* with intensity *I*_0_, it attenuates and its intensity is reduced to *I* [25]. Another important property when it comes to investigating any radiation-shielding material is the half-value layer which can be found using Equations (2) and (3):

(2) $$HVL=\frac{\ln2}{\mu }$$

(3) $$\mu_{m}=\mu /\rho$$

where *μ* is the linear attenuation coefficient and *ρ* is the material density [37,38,39,40].

The total atomic cross-section can be calculated using Equation (4), where *N_A_* is Avogadro’s number and *A_i_* is the atomic weight of an element of the compound, while the total electronic cross-section for the element is given by Equation (5) [42,43,44,45]:

(4) $$\sigma_{t,a}=\frac{\mu_{m}}{N_{A}\sum_{i}^{n}\left(w_{i}/A_{i}\right)}$$

(5) $$\sigma_{t,el}=\frac{1}{N_{A}}\sum_{i}^{n}\frac{f_{i}A_{i}}{Z_{i}}{\left(\mu_{mt}\right)}_{i}$$

where *f_i_* is the number of atoms of the element *i* relative to the total number of atoms of all elements in the compound and *Z_i_* is the atomic number of the *i*th element in the compound. The effective atomic number (*Z_eff_*) of the compound can be found from the ratio between the total atomic cross-section and the total electronic cross-section using Equation (6), and the effective electron density is given by Equation (7) [42,43,44,45]:

(6) $$Z_{eff}=\frac{\sigma_{t,a}}{\sigma_{t,el}}$$

(7) $$N_{eff}=\frac{\mu_{m}}{\sigma_{t,el}}$$

All these important parameters were investigated theoretically in this work on the PLA–PHB blends in the studied energy range.

### 3.2. Neutron Attenuation 

The probability of neutron reactions with any material is expressed by the neutron-removing cross-section (Σ*_R_*), which is given by Equation (8) [46,47,48]:

(8) $$\Sigma_{R}={\sum_{i}\rho_{i}\left(\Sigma_{R}/\rho \right)}_{i}$$

where *ρ_i_* is the partial density and Σ*_R_*/*ρ* is the mass removal cross-section, which can be calculated using Equation (9) for any compound where *A* is the atomic weight and *Z* is the atomic number [46,47,48].

(9) ΣRρ=0.206A−13Z−0.294

The fast neutron removal cross-section can be found for any element using Equations (10) and (11) [41,42,43]:

(10) $$\Sigma_{R}=0.190Z^{-0.743}\;\;\;if\;Z\leq 8$$

(11) $$\Sigma_{R}=0.125Z^{-0.565}\;\;\;if\;Z>8$$

Equation (12) gives the mean free Path (*λ*), which is the distance that the neutron travels without interaction, and Equation (13) gives the HVL [46,47,48].

(12) $$\lambda =\frac{1}{\Sigma_{R}}$$

(13) $$HVL=\frac{\ln2}{\Sigma_{R}}$$

These important neutron-shielding parameters were also investigated theoretically in the studied energy range for the PLA–PHB blends.

## 4. Methods

**Figure 2:**
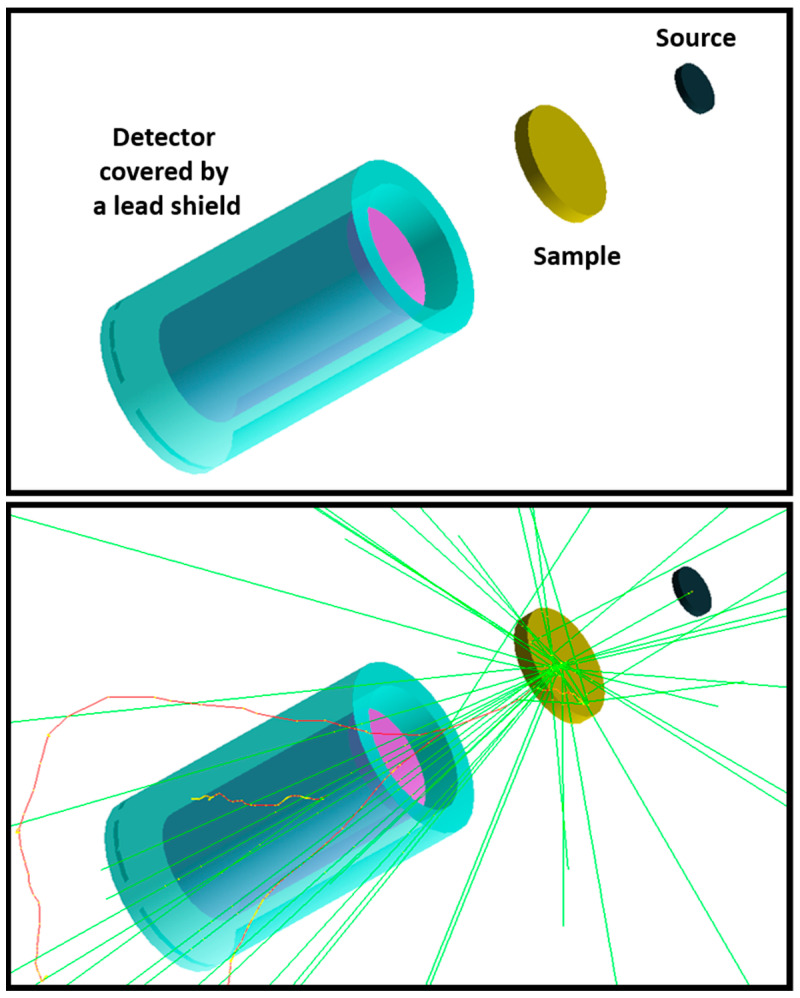
Visualization of the developed Geant4 code (green lines in the bottom image represent the photons or neutrons, and the red lines represent negatively charged particles).

Geant4, a powerful Monte Carlo toolkit, version 11.02, is utilized in nuclear physics, nuclear engineering and medical physics [49]. Here, it was used to investigate the studied blends’ photon- and neutron-shielding properties. A Geant4 code was developed to study the interactions of both gammas and neutrons in an energy range between 10 keV and 20 MeV. A source was placed in front of the investigated sample shooting monoenergetic gamma and neutron particles in the direction of the sample followed by a detector covered by a lead shield. The attenuation of both gammas and neutrons was measured by determining the ratio between the number of particles reaching the detector with and without the sample. For each energy, 1,000,000 monoenergetic particles were emitted in a direction perpendicular to the sample. Figure 2 shows a screenshot of the Geant4 simulation code used in this study. In order to validate the results obtained by the Geant4 code, the photon-shielding properties were compared to those found by EpiXS, which is a Windows-based program for photon attenuation, dosimetry and shielding based on the EPICS2017 and EPDL9 databases that can obtain the photon cross-section data for any sample [50]. Root, version 6.10/04, software was used to plot the figures presented in this article [51]. 

## 5. PLA Photon-Shielding Properties and Validation of Geant4 Code Results

### 5.1. PLA Photon-Shielding Capbilities

The photon-shielding properties of PLA were investigated in a wide energy range from 10 keV to 20 MeV and compared to those found by EpiXS using Equation (14) in order to validate the results obtained by Geant4 (Table 2).

(14) $$∆\%=\frac{\left(Geant4\;result-EpiXS\;result\right)}{EpiXS\;result}\times 100$$

The results obtained from Geant4 show excellent agreement with those from EpiXS as the percentage differences between them were less than 1% in the case of the mass attenuation coefficients, with an average of 0.14% for the whole energy range. The percentage differences in the case of the estimates of HVLs were less than 1% as well, with an average of −0.14% for the whole energy range. In the case of the results of the effective atomic numbers and effective neutron densities, the results were in good agreement and had an average percentage difference of −1.57% and −1.61%, respectively; at lower energies, the differences between the results obtained from Geant4 and EpiXS were greater but did not exceed 10%. These results confirm that the Geant4 code is reliable in estimating the photon-shielding capabilities of any material.

### 5.2. PLA Neutron-Shielding Capabilities

In order to verify the Geant4 results in the case of the neutron-shielding properties of simulated materials, the fast neutron removal cross-section of PLA was estimated using Equations (9)–(11), as shown in Table 3. 

The fast neutron removal cross-section of PLA was simulated using Geant4 and was equal to 0.060894 cm^−1^ which is in very good agreement with the neutron removal cross-section found in Table 3, indicating that Geant4 can reliably predict the neutron-shielding abilities of any material. The neutron removal cross-sections and half-value layers of PLA were investigated using Geant4 in an energy range from 10 keV to 20,000 keV, as shown in Table 4.

As the results obtained by Geant4 were validated and the photon- and neutron-shielding properties of PLA were verified, we can estimate the effect of blending PLA with PHB on the shielding properties and the effect of doping those blends with different metal oxides.

## 6. Effect of PLA Blending with PHB on the PLA-Shielding Properties 

### 6.1. PLA–PHB Blends’ Photon-Shielding Capabilities

**Figure 3:**
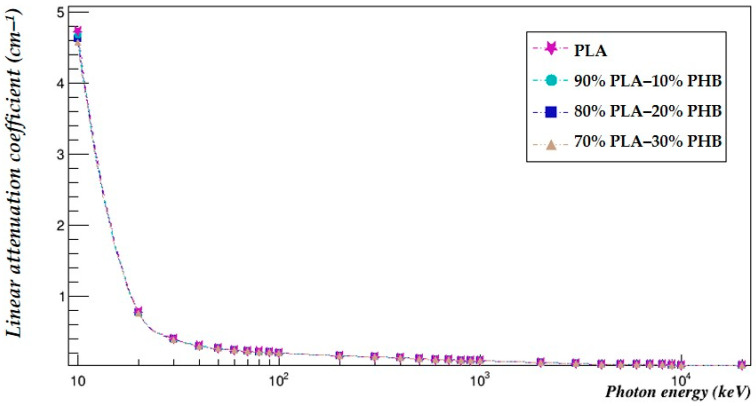
The linear attenuation coefficients of the investigated blends.

The photon-shielding properties of all the investigated blends were compared using Geant4 in the same energy range (Table 5). The linear attenuation coefficients of all the studied blends were plotted against that of PLA to more easily visualize the comparison (Figure 3).

### 6.2. PLA–PHB Blends’ Neutron-Shielding Capabilities

**Figure 4:**
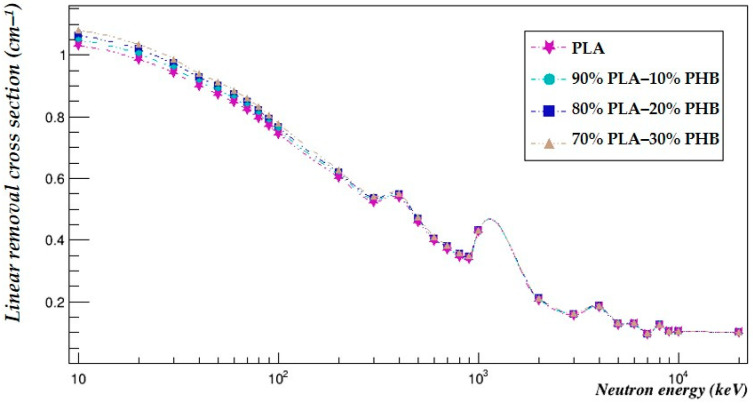
The linear removal cross-sections of the investigated blends.

The neutron-shielding properties of the investigated blends were compared using Geant4 in the same energy range (Table 6). The linear removal cross-sections of all the studied blends were plotted against that of PLA to more easily visualize the comparison (Figure 4).

## 7. Effect of Metal Oxide Doping on PLA–PHB Blends’ Shielding Properties 

**Figure 5:**
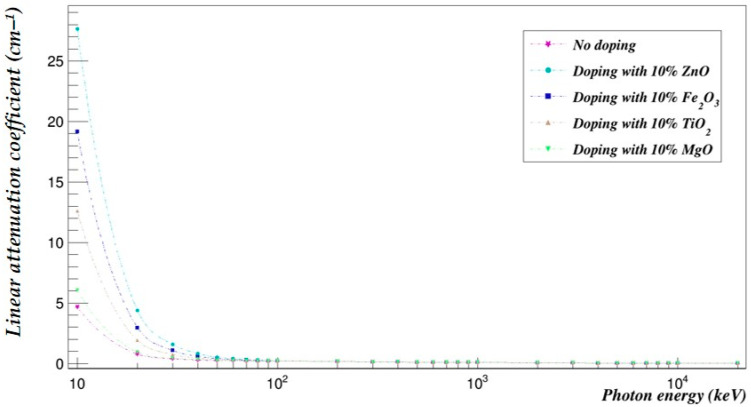
The photon linear attenuation coefficients of the PLA–PHB (90–10%) blend doped with 10% metal oxide.

**Figure 6:**
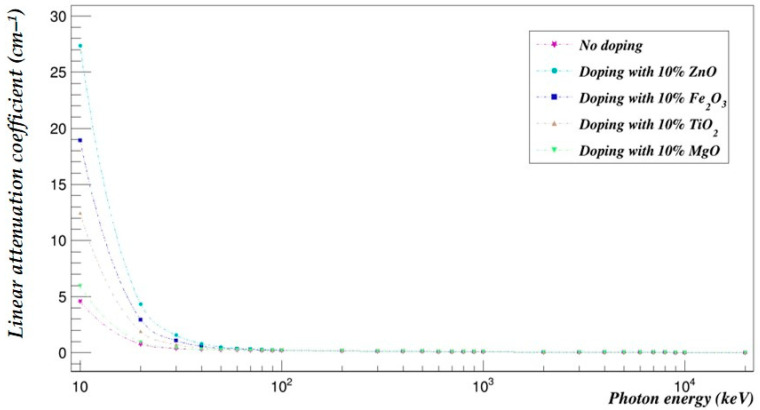
The photon linear attenuation coefficients of the PLA–PHB (70–30%) blend doped with 10% metal oxide.

**Figure 7:**
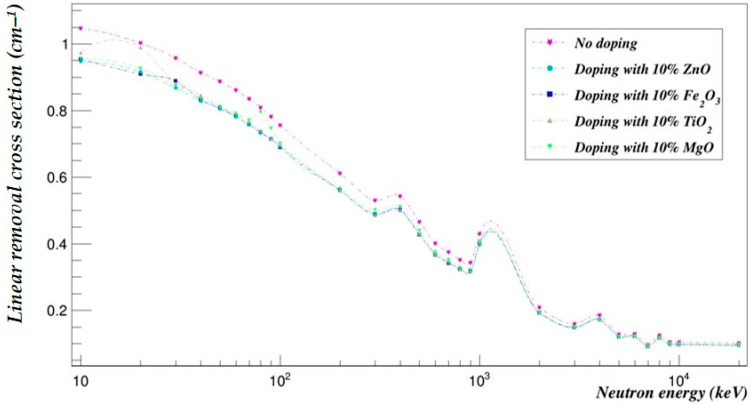
The neutron linear removal cross-sections of the PLA–PHB (90–10%) blend doped with 10% metal oxide.

**Figure 8:**
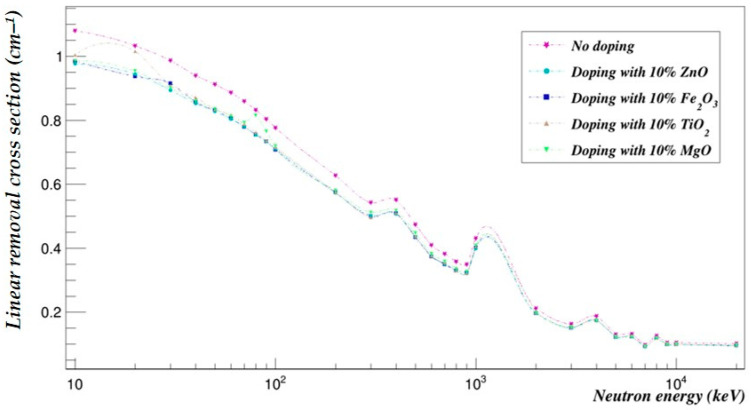
The neutron linear removal cross-sections of the PLA–PHB (70–30%) blend doped with 10% metal oxide.

The photon- and neutron-shielding properties of the 90–10% and the 70–30% blends doped with 10% of selected metal oxides (ZnO, Fe_2_O_3_, TiO_2_ and MgO) were compared using Geant4 in the same energy range (Figure 5, Figure 6, Figure 7 and Figure 8). 

### 7.1. Effect of Metal Oxide Doping on the PLA–PHB Blends’ Photon-Shielding Capabilities

Figure 5 and Figure 6 represent the photon-shielding properties of the 90–10% and the 70–30% blends doped with 10% of selected metal oxides (ZnO, Fe_2_O_3_, TiO_2_ and MgO).

### 7.2. Effect of Metal Oxide Doping on the PLA–PHB Blends’ Neutron-Shielding Capabilities

Figure 7 and Figure 8 represent the neutron-shielding properties of the 90–10% and the 70–30% blends doped with 10% of selected metal oxides (ZnO, Fe_2_O_3_, TiO_2_ and MgO).

## 8. Discussion

The results show that the mass attenuations of the 90–10% PLA–PHB blend decreased compared to those of PLA in an energy range of 10 to 40 keV by 0.08% to 0.75% and at an energy of 20,000 keV by 0.04%. In addition, the mass attenuations of the blend with 80–20% fractions decreased from 0.15% to 1.5% at energies ranging from 10 keV to 40 keV and by 0.07% at 20,000 keV. On the other hand, the mass attenuations of the blend with 70–30% fractions decreased from 0.23% to 2.25% at energies ranging from 10 keV to 40 keV and by 0.11% at 20,000 keV. Meanwhile, at all the other studied energies, the mass attenuation coefficients increased by an average of 0.1% for the 90–10% blend, 0.2% for the 80–20% blend and 0.3% for the 70–30% blend.

The linear attenuation coefficients of the 90–10%, 80–20% and 70–30% blends decreased by an average of 0.38%, 0.75% and 1.13%, while the HVL increased by an average of 0.38%, 0.76% and 1.15%, respectively.

The blending decreased the ability of PLA to shield against photons especially at low energies and very-high-energy photons. In addition, the higher the fraction of PHB added to the blend, the lower the photon-shielding capability of the blend.

The results show that the neutron removal cross-sections were increased by an average of 1.51% for the 90–10% blend, 3.01% for the 80–20% blend and 4.53% for the 70–30% blend.

The neutron half-value layers were decreased by an average of 1.09% for the 90–10% blend, 2.13% for the 80–20% blend and 3.15% for the 70–30% blend.

The blending increased the capability of PLA to attenuate neutrons in the whole studied energy range, and the ability of the blends to shield against neutrons increased with the fraction of PHB added to the blend. 

The results clearly show that the neutron-shielding capabilities are affected by the blending of the biopolymers more than the photon-shielding capabilities. These results also show the influence of the molecular weight of polymers on photon- and neutron-shielding abilities.

The photon-shielding abilities of the 90–10% and 70–30% blends were increased in the studied energy range especially at low photon energies. ZnO had the most impact on the attenuation coefficients of the 90–10% blend with an average increase of 59.91%, followed by Fe_2_O_3_, which increased the photon-shielding abilities by an average of 35.79% over the investigated energy range; then TiO_2_, which increased the attenuation coefficients by an average of 18.49%; and finally, MgO, which increased them by an average of 2.64%. On the other hand, the 70–30% blend’s photon attenuation coefficients were increased by an average of 60.39% when it was doped with 10% ZnO, 36.08% with Fe_2_O_3_, 18.64% with TiO_2_ and 2.67% with MgO.

The neutron-shielding capabilities of both the 90–10% and 70–30% blends were decreased by an average of 14.10% and 7.90% when doped with ZnO, 13.94% and 7.73% with Fe_2_O_3_, 13.23% and 6.98% with TiO_2_ and 12.65% and 6.38% with MgO, respectively.

## 9. Conclusions

The blending of polymers could enhance many properties that could be used in many applications. One of the properties affected by polymer blending is the radiation-shielding capabilities. In this work, the photon- and neutron-shielding abilities of PLA–PHB blends were simulated using the Monte Carlo toolkit Geant4. 

The results show the following:

- The photon-shielding properties decreased when blending PHB with PLA, while the neutron-shielding abilities were improved by blending.
- Doping the PLA–PHB blends with metal oxides enhanced the photon-shielding capabilities of the studied blends and slightly reduced their neutron-shielding capabilities.
- Geant4 is a very reliable tool which can be used to simulate the interactions of any radiation type with any material and, hence, evaluate the shielding capabilities of different materials.

The use and application of the blends should be considered when choosing the right type and fraction of polymers to blend. On the other hand, doping with metal oxides could affect the shielding properties of different polymer blends and the type of dopants must be considered based on the desired applications because they could affect the photon- and neutron-shielding abilities in different ways. 

## Figures and Tables

**Table 1:** Element weight fractions in PLA and PHB.

Element	PLA	PHB
**H (Hydrogen)**	0.05595	0.07025
**C (Carbon)**	0.50001	0.55806
**O (Oxygen)**	0.44404	0.37169

**Table 2:** Photon-shielding properties of PLA.

**Energy (keV)**	**Geant4**	**EpiXS**	**∆%**	**Geant4**	**EpiXS**	**∆%**
	***μ_m_* (cm^2^/g)**			**HVL (cm)**		
**10**	3.8155	3.8541	−1.00%	0.1465	0.1450	1.01%
**20**	0.6288	0.6269	0.31%	0.8890	0.8917	−0.31%
**30**	0.3181	0.3162	0.59%	1.7575	1.7676	−0.57%
**40**	0.2387	0.2382	0.21%	2.3417	2.3468	−0.22%
**50**	0.2088	0.2071	0.80%	2.6777	2.6987	−0.78%
**60**	0.1919	0.1906	0.69%	2.9126	2.9330	−0.70%
**70**	0.1813	0.1799	0.80%	3.0830	3.1078	−0.80%
**80**	0.1728	0.1723	0.30%	3.2349	3.2445	−0.30%
**90**	0.1666	0.1660	0.37%	3.3555	3.3676	−0.36%
**100**	0.1610	0.1610	0.01%	3.4716	3.4723	−0.02%
**200**	0.1291	0.1299	−0.65%	4.3315	4.3018	0.69%
**300**	0.1123	0.1126	−0.28%	4.9786	4.9637	0.30%
**400**	0.1009	0.1008	0.15%	5.5393	5.5482	−0.16%
**500**	0.0924	0.0919	0.49%	6.0517	6.0795	−0.46%
**600**	0.0855	0.0850	0.55%	6.5344	6.5739	−0.60%
**700**	0.0799	0.0794	0.66%	6.9918	7.0422	−0.72%
**800**	0.0753	0.0747	0.86%	7.4254	7.4871	−0.82%
**900**	0.0713	0.0706	1.00%	7.8454	7.9183	−0.92%
**1000**	0.0676	0.0671	0.75%	8.2726	8.3311	−0.70%
**2000**	0.0469	0.0468	0.13%	11.9291	11.9343	−0.04%
**3000**	0.0375	0.0376	−0.24%	14.9209	14.8707	0.34%
**4000**	0.0320	0.0322	−0.56%	17.4414	17.3711	0.40%
**5000**	0.0285	0.0286	−0.45%	19.6034	19.5246	0.40%
**6000**	0.0260	0.0261	−0.50%	21.4727	21.3925	0.37%
**7000**	0.0242	0.0243	−0.29%	23.1003	23.0326	0.29%
**8000**	0.0228	0.0229	−0.27%	24.5232	24.4511	0.29%
**9000**	0.0217	0.0217	−0.20%	25.7737	25.7091	0.25%
**10,000**	0.0208	0.0208	−0.19%	26.8773	26.8239	0.20%
**20,000**	0.0169	0.0169	0.00%	33.0325	33.0776	−0.14%
**Energy (keV)**	**Geant4**	**EpiXS**	**∆%**	**Geant4**	**EpiXS**	**∆%**
	***Z_eff_***			***N_eff_* (×10^23^)**		
**10**	6.107	6.121	−0.22%	4.700	4.603	2.10%
**20**	5.486	5.880	−6.71%	4.190	4.423	−5.26%
**30**	4.897	5.392	−9.19%	3.710	4.056	−8.52%
**40**	4.583	4.938	−7.19%	3.460	3.714	−6.83%
**50**	4.470	4.673	−4.34%	3.360	3.515	−4.40%
**60**	4.395	4.523	−2.83%	3.300	3.402	−3.00%
**70**	4.360	4.437	−1.74%	3.270	3.337	−2.02%
**80**	4.314	4.384	−1.59%	3.240	3.297	−1.73%
**90**	4.302	4.349	−1.09%	3.230	3.271	−1.26%
**100**	4.277	4.326	−1.13%	3.210	3.254	−1.34%
**200**	4.219	4.261	−0.99%	3.160	3.205	−1.41%
**300**	4.231	4.251	−0.48%	3.170	3.198	−0.86%
**400**	4.248	4.247	0.01%	3.180	3.195	−0.46%
**500**	4.260	4.246	0.33%	3.190	3.194	−0.11%
**600**	4.266	4.245	0.49%	3.200	3.193	0.22%
**700**	4.271	4.245	0.62%	3.200	3.193	0.23%
**800**	4.275	4.245	0.72%	3.200	3.192	0.24%
**900**	4.279	4.244	0.82%	3.210	3.192	0.56%
**1000**	4.270	4.244	0.61%	3.200	3.192	0.25%
**2000**	4.249	4.255	−0.13%	3.190	3.200	−0.31%
**3000**	4.252	4.282	−0.71%	3.190	3.221	−0.96%
**4000**	4.273	4.316	−1.01%	3.200	3.246	−1.43%
**5000**	4.299	4.352	−1.22%	3.230	3.273	−1.32%
**6000**	4.327	4.388	−1.38%	3.250	3.300	−1.52%
**7000**	4.358	4.422	−1.45%	3.270	3.326	−1.68%
**8000**	4.385	4.454	−1.55%	3.290	3.350	−1.79%
**9000**	4.413	4.485	−1.60%	3.320	3.373	−1.57%
**10,000**	4.441	4.513	−1.59%	3.340	3.394	−1.60%
**20,000**	4.662	4.705	−0.91%	3.510	3.538	−0.80%

**Table 3:** Fast neutron removal cross-section of PLA.

Element	Weight Fraction	Σ*_R_*/*ρ* (cm^2^/g)	Partial Density (g/cm^3^)	Σ*_R_* (cm^−1^)
H (Hydrogen)	0.0559	0.19000	0.0694	0.013181
C (Carbon)	0.5000	0.05019	0.6200	0.031118
O (Oxygen)	0.4440	0.04053	0.5506	0.022316
**Total fast neutron removal cross-section** ** = 0.066616**				

**Table 4:** Neutron-shielding properties of PLA.

Energy (keV)	Σ*_R_* (cm^2^/g)	*HVL* (cm)
**10**	0.8310	0.6726
**20**	0.7956	0.7026
**30**	0.7603	0.7353
**40**	0.7248	0.7713
**50**	0.7043	0.7937
**60**	0.6837	0.8177
**70**	0.6631	0.8430
**80**	0.6424	0.8702
**90**	0.6216	0.8993
**100**	0.6008	0.9305
**200**	0.4864	1.1492
**300**	0.4222	1.3239
**400**	0.4345	1.2865
**500**	0.3714	1.5053
**600**	0.3199	1.7476
**700**	0.2989	1.8702
**800**	0.2803	1.9946
**900**	0.2742	2.0390
**1000**	0.3456	1.6176
**2000**	0.1660	3.3680
**3000**	0.1269	4.4060
**4000**	0.1478	3.7826
**5000**	0.1023	5.4648
**6000**	0.1038	5.3863
**7000**	0.0766	7.2967
**8000**	0.0997	5.6095
**9000**	0.0832	6.7205
**10,000**	0.0830	6.7310
**20,000**	0.0816	6.8480

**Table 5:** Photon-shielding properties of PLA–PHB blends.

**Energy (keV)**	***μ_m_* (cm^2^/g)**		
	**90% PLA–10% PHB**	**80% PLA–20% PHB**	**70% PLA–30% PHB**
**10**	3.7870	3.7584	3.7298
**20**	0.6255	0.6222	0.6190
**30**	0.3173	0.3165	0.3157
**40**	0.2385	0.2383	0.2382
**50**	0.2088	0.2088	0.2088
**60**	0.1920	0.1921	0.1922
**70**	0.1815	0.1816	0.1817
**80**	0.1730	0.1731	0.1733
**90**	0.1668	0.1669	0.1671
**100**	0.1612	0.1614	0.1616
**200**	0.1292	0.1294	0.1295
**300**	0.1124	0.1126	0.1127
**400**	0.1010	0.1012	0.1013
**500**	0.0925	0.0926	0.0927
**600**	0.0857	0.0858	0.0859
**700**	0.0801	0.0802	0.0803
**800**	0.0754	0.0755	0.0756
**900**	0.0713	0.0714	0.0715
**1000**	0.0677	0.0678	0.0678
**2000**	0.0469	0.0470	0.0470
**3000**	0.0375	0.0376	0.0376
**4000**	0.0321	0.0321	0.0322
**5000**	0.0285	0.0286	0.0286
**6000**	0.0261	0.0261	0.0261
**7000**	0.0242	0.0242	0.0243
**8000**	0.0228	0.0228	0.0228
**9000**	0.0217	0.0217	0.0217
**10,000**	0.0208	0.0208	0.0208
**20,000**	0.0169	0.0169	0.0169
**Energy (keV)**	***Z_eff_***		
	**90% PLA–10% PHB**	**80% PLA–20% PHB**	**70% PLA–30% PHB**
**10**	1.466	1.455	1.444
**20**	1.311	1.304	1.297
**30**	1.163	1.161	1.158
**40**	1.085	1.084	1.084
**50**	1.057	1.057	1.057
**60**	1.038	1.038	1.039
**70**	1.029	1.030	1.031
**80**	1.018	1.019	1.020
**90**	1.015	1.016	1.017
**100**	1.009	1.010	1.010
**200**	0.995	0.996	0.997
**300**	0.998	0.999	1.000
**400**	1.002	1.003	1.004
**500**	1.005	1.006	1.007
**600**	1.006	1.007	1.009
**700**	1.007	1.008	1.010
**800**	1.008	1.009	1.011
**900**	1.009	1.010	1.012
**1000**	1.007	1.008	1.010
**2000**	1.002	1.003	1.005
**3000**	1.003	1.004	1.005
**4000**	1.008	1.009	1.010
**5000**	1.014	1.015	1.016
**6000**	1.021	1.022	1.023
**7000**	1.029	1.030	1.030
**8000**	1.035	1.036	1.037
**9000**	1.042	1.043	1.043
**10,000**	1.049	1.050	1.050
**20,000**	1.103	1.103	1.102
**Energy (keV)**	***N_eff_* (*×*10^23^)**		
	**90% PLA–10% PHB**	**80% PLA–20% PHB**	**70% PLA–30% PHB**
**10**	4.67	4.63	4.60
**20**	4.17	4.15	4.13
**30**	3.70	3.69	3.69
**40**	3.45	3.45	3.45
**50**	3.36	3.36	3.36
**60**	3.30	3.31	3.31
**70**	3.28	3.28	3.28
**80**	3.24	3.24	3.25
**90**	3.23	3.23	3.24
**100**	3.21	3.22	3.22
**200**	3.17	3.17	3.17
**300**	3.18	3.18	3.18
**400**	3.19	3.19	3.20
**500**	3.20	3.20	3.21
**600**	3.20	3.21	3.21
**700**	3.21	3.21	3.21
**800**	3.21	3.21	3.22
**900**	3.21	3.22	3.22
**1000**	3.20	3.21	3.21
**2000**	3.19	3.19	3.20
**3000**	3.19	3.20	3.20
**4000**	3.21	3.21	3.22
**5000**	3.23	3.23	3.24
**6000**	3.25	3.25	3.26
**7000**	3.27	3.28	3.28
**8000**	3.30	3.30	3.30
**9000**	3.32	3.32	3.32
**10,000**	3.34	3.34	3.34
**20,000**	3.51	3.51	3.51

**Table 6:** Neutron-shielding properties of PLA–PHB blends.

**Energy (keV)**	**Σ*_R_* (cm^2^/g)**		
	**90% PLA–10% PHB**	**80% PLA–20% PHB**	**70% PLA–30% PHB**
**10**	0.8480	0.8646	0.8816
**20**	0.8117	0.8275	0.8436
**30**	0.7753	0.7904	0.8055
**40**	0.7391	0.7533	0.7674
**50**	0.7180	0.7317	0.7454
**60**	0.6968	0.7101	0.7233
**70**	0.6758	0.6885	0.7012
**80**	0.6546	0.6668	0.6790
**90**	0.6332	0.6449	0.6566
**100**	0.6120	0.6231	0.6343
**200**	0.4949	0.5034	0.5119
**300**	0.4292	0.4362	0.4431
**400**	0.4397	0.4447	0.4498
**500**	0.3764	0.3815	0.3866
**600**	0.3247	0.3296	0.3344
**700**	0.3035	0.3080	0.3126
**800**	0.2845	0.2887	0.2929
**900**	0.2779	0.2816	0.2854
**1000**	0.3478	0.3500	0.3522
**2000**	0.1685	0.1711	0.1736
**3000**	0.1288	0.1308	0.1328
**4000**	0.1495	0.1511	0.1528
**5000**	0.1038	0.1052	0.1067
**6000**	0.1049	0.1060	0.1071
**7000**	0.0777	0.0787	0.0798
**8000**	0.1009	0.1022	0.1035
**9000**	0.0841	0.0849	0.0858
**10,000**	0.0838	0.0846	0.0854
**20,000**	0.0820	0.0823	0.0828
**Energy (keV)**	***HVL* (cm)**		
	**90% PLA–10% PHB**	**80% PLA–20% PHB**	**70% PLA–30% PHB**
**10**	0.6619	0.6518	0.6419
**20**	0.6915	0.6810	0.6707
**30**	0.7239	0.7129	0.7025
**40**	0.7594	0.7481	0.7373
**50**	0.7817	0.7702	0.7591
**60**	0.8054	0.7936	0.7823
**70**	0.8305	0.8185	0.8070
**80**	0.8574	0.8452	0.8334
**90**	0.8863	0.8738	0.8618
**100**	0.9171	0.9043	0.8921
**200**	1.1341	1.1194	1.1054
**300**	1.3077	1.2920	1.2769
**400**	1.2766	1.2671	1.2580
**500**	1.4910	1.4772	1.4638
**600**	1.7284	1.7099	1.6919
**700**	1.8496	1.8297	1.8103
**800**	1.9730	1.9520	1.9316
**900**	2.0198	2.0010	1.9829
**1000**	1.6138	1.6101	1.6064
**2000**	3.3305	3.2944	3.2594
**3000**	4.3562	4.3084	4.2621
**4000**	3.7555	3.7293	3.7038
**5000**	5.4091	5.3548	5.3020
**6000**	5.3519	5.3174	5.2852
**7000**	7.2265	7.1584	7.0925
**8000**	5.5603	5.5124	5.4659
**9000**	6.6774	6.6354	6.5947
**10,000**	6.6943	6.6585	6.6235
**20,000**	6.8481	6.8511	6.8307

## Data Availability

The data presented in this study are available on request from the corresponding author.
